# Acute Superior Vena Cava Obstruction Mimicking Aortic Dissection: A Case of Primary Mediastinal Large B-cell Lymphoma

**DOI:** 10.7759/cureus.102581

**Published:** 2026-01-29

**Authors:** Sanjana Ulfat Chadni, Tanvir Ahmed, Sabiha Jahan, Muhammad Sarwar, Faaraan Bangash

**Affiliations:** 1 Acute Medicine, Northampton General Hospital NHS Trust, Northampton, GBR; 2 Public Health, Baylor University, Waco, USA; 3 Medicine, Mugda Medical College and Hospital, Dhaka, BGD

**Keywords:** aortic dissection mimic, back pain radiating to chest, ct aortogram, emergency medicine, primary mediastinal large b-cell lymphoma, superior vena cava obstruction

## Abstract

Superior vena cava (SVC) obstruction typically presents with facial swelling, upper-limb edema, and dyspnea due to impaired venous drainage from the upper body. Acute presentations are uncommon and may differ markedly from the classic clinical picture. We report the case of a 27-year-old woman who presented with sudden back pain radiating to the chest. This was accompanied by coldness, numbness, and discoloration of the left upper limb, initially raising concern for acute aortic dissection. A CT aortogram excluded arterial pathology. However, it revealed a large anterior mediastinal mass compressing the SVC, consistent with acute SVC obstruction. Laboratory evaluation showed elevated lactate dehydrogenase and CA-125 levels. Histopathological examination confirmed a diagnosis of primary mediastinal large B-cell lymphoma. The patient experienced rapid symptomatic improvement following corticosteroid therapy. Subsequent treatment with R-CHOP chemotherapy (rituximab, cyclophosphamide, doxorubicin, vincristine, and prednisolone) resulted in a marked clinical and radiological response. This case highlights an atypical, pain-dominant presentation of acute SVC obstruction mimicking acute aortic dissection. It emphasizes the importance of considering venous etiologies early to avoid diagnostic delay in patients presenting with acute chest pain and limb vascular abnormalities.

## Introduction

Superior vena cava (SVC) obstruction refers to impaired venous return from the head, neck, upper extremities, and upper thorax due to narrowing or blockage of the SVC [1]. It is most commonly caused by external compression, thrombosis, or intraluminal invasion, with malignancy accounting for the majority of cases [2]. Patients typically present with manifestations of elevated venous pressure above the obstruction, including facial swelling or plethora, upper-limb edema, distended neck veins, dyspnea, cough, headache, and a sensation of fullness in the head [1].

Chronic SVC obstruction usually develops gradually over weeks to months, allowing collateral venous pathways to form [3]. As a result, symptoms may be relatively mild despite significant anatomic narrowing [3]. Typical chronic features include progressive facial puffiness, upper-limb swelling, prominent chest-wall collaterals, and positional worsening of symptoms when lying flat [1].

In contrast, acute SVC obstruction evolves over hours to days and lacks the benefit of established collateral drainage [4]. This leads to rapid, progressive, and often dramatic symptoms such as sudden facial and arm swelling, marked venous engorgement, dyspnea, cyanosis, and headache. Acute presentations may also include severe chest or back pain, occasionally mimicking arterial emergencies such as aortic dissection [1,4]. Such pain-dominant presentations are uncommon and may divert clinicians toward arterial diagnoses, increasing the risk of misdiagnosis or inappropriate management.

We describe a case of mediastinal lymphoma presenting with acute SVC obstruction that clinically resembled an aortic dissection, highlighting the importance of considering venous etiologies in patients with sudden chest or back pain and upper-extremity vascular findings.

## Case presentation

A 27-year-old woman with no significant past medical history presented with sudden-onset, sharp back pain radiating to the chest, rated 6/10 in intensity, which abruptly awakened her from sleep. She also described coldness, numbness, and dusky discoloration of the left upper limb, while the right upper limb remained normal.

On examination, her blood pressure was 140/59 mmHg, with no interarm difference. The left radial pulse was delayed, and the capillary refill time exceeded seven seconds. Notably, there was no facial plethora, no upper-limb swelling, and no distended neck veins. Cardiovascular, respiratory, and systemic examinations were otherwise unremarkable. Throughout the initial assessment and diagnostic workup, the patient remained hemodynamically stable, with no evidence of cardiovascular compromise.

**Figure 1:**
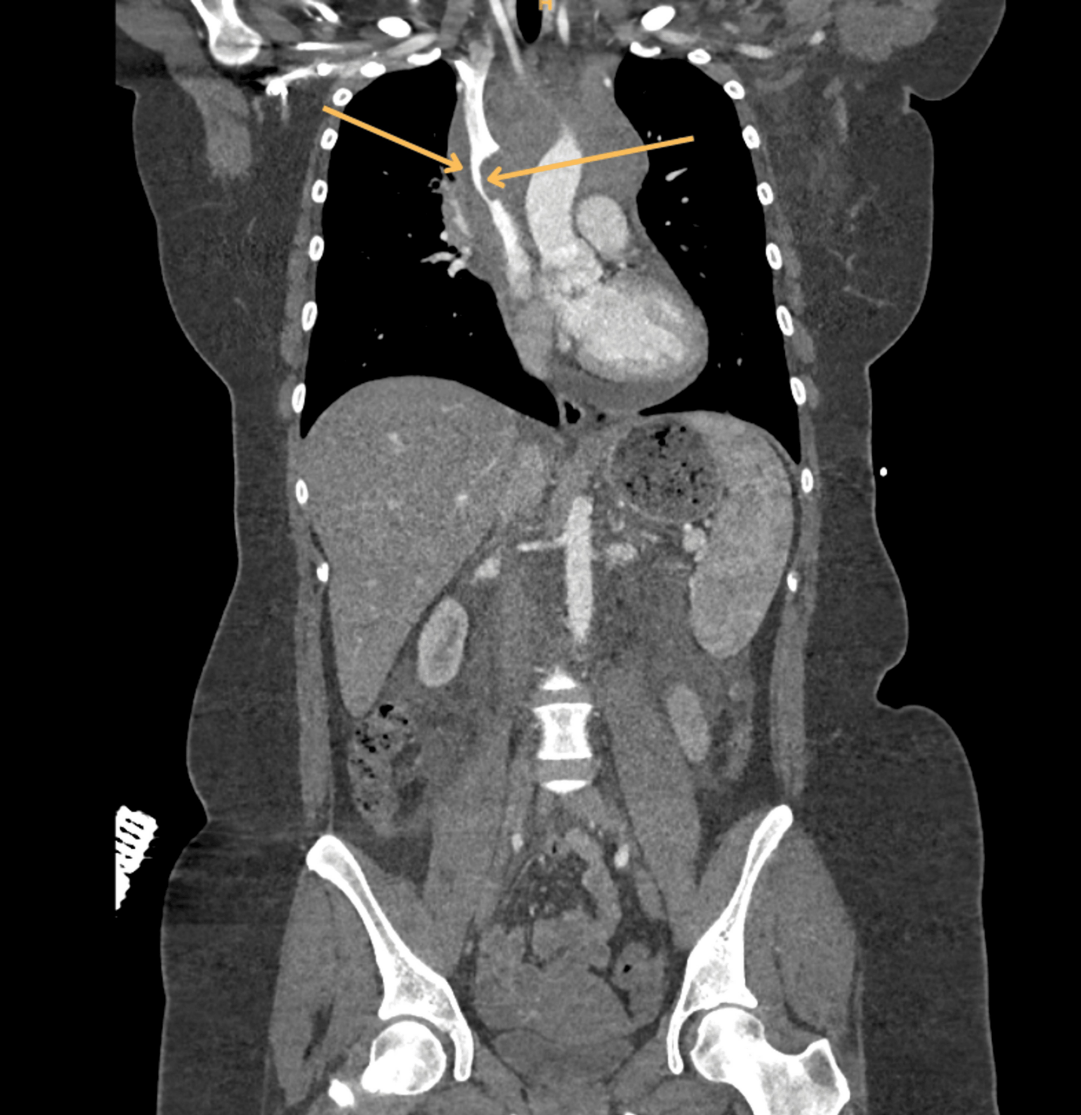
Coronal reformatted contrast-enhanced CT aortogram demonstrating a large mediastinal mass measuring 13 × 11 × 10 cm, encasing the aortic arch and causing marked extrinsic compression and narrowing of the SVC (arrows), consistent with SVC obstruction CT: computed tomography, SVC: superior vena cava

**Figure 2:**
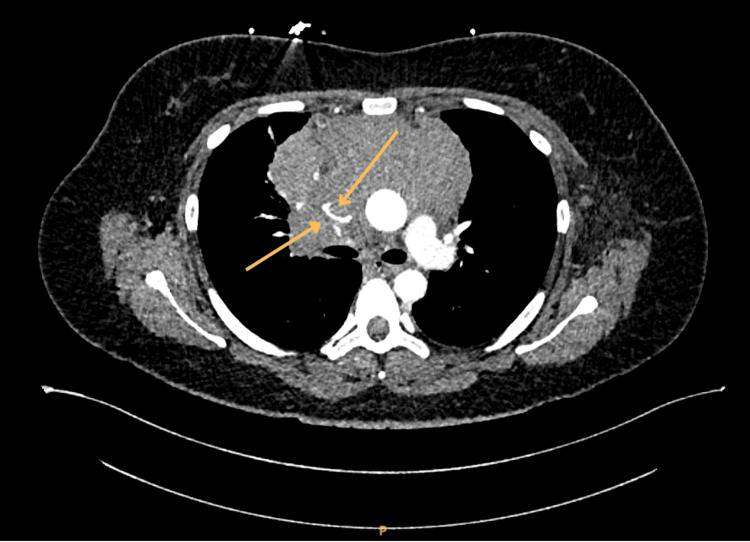
Axial CT aortogram showing a bulky anterior mediastinal mass with marked compression of the SVC (arrows), consistent with SVC obstruction CT: computed tomography, SVC: superior vena cava

Given the combination of acute chest/back pain and asymmetric upper-limb vascular findings, an acute aortic dissection was initially suspected. An urgent CT aortogram excluded dissection but demonstrated a large anterior mediastinal mass compressing the SVC (Figures 1-2).

Initial laboratory investigations demonstrated anemia, with elevated lactate dehydrogenase and D-dimer levels, normal serum troponin-I, and normal renal and liver function tests (Table 1).

**Table 1:** Initial laboratory findings demonstrating anemia with elevated LDH and D-dimer levels. Renal and liver function tests and serum troponin-I were within normal limits LDH: lactate dehydrogenase

Parameter	Result	Reference range
Hemoglobin	106 g/L	120-150
Hematocrit	33%	36-46
White cell count	7.9 x 10*9/L	4.0-10.0
Mean cell volume	83 fL	83-101
Mean cell hemoglobin	26.0 pg	27-32
Neutrophil count	6.57 x 10*9/L	1.8-7.4
Lymphocytes	0.75 x 10*9/L	1.1-3.5
Platelet count	465 x 10*9/L	150-400
C-reactive protein	138 mg/L	0-5
Sodium	137 mmol/L	133-146
Potassium	4.6 mmol/L	3.5-5.3
Urea	2.4 mmol/L	2.5-7.8
Creatinine	62 umol/L	45-84
Estimated glomerular filtration rate	>90 ml/min	-
Total protein	68 g/L	60-80
Albumin	39 g/L	35-50
Corrected calcium	2.42 mmol/L	2.20-2.60
Total bilirubin	9 umol/L	0-21
Alkaline phosphatase	140 IU/L	30-130
Alanine aminotransferase	42 IU/L	0-35
International normalized ratio	1.0 ratio	0.8-1.2
Activated partial thromboplastin time	24 secs	22-30
D-dimer	1356 ng/mL	-
Magnesium	0.78 mmol/L	0.70-1.00
Lactate dehydrogenase	549 IU/L	136-214
Troponin	<13 ng/L	<15

After consultation with oncology and hematology, tumor markers were requested to evaluate for malignancy; CA-125 and lactate dehydrogenase were elevated, and IgA and IgM were normal on protein electrophoresis (Table 2).

**Table 2:** Tumor marker evaluation demonstrating elevated CA-125 with other markers within normal reference ranges AFP: alpha-fetoprotein, CEA: carcinoembryonic antigen, HCG: human chorionic gonadotropin, LDH: lactate dehydrogenase, Ig: immunoglobulin

Parameter	Result	Reference range
CA 15-3	17 KU/L	0-30
CA 19-9	<9 KU/L	0-34
Ca125	70 KU/L	0-35
AFP	<2 KU/L	0-6
CEA	2.0 µg/L	0.0-4.7
Total HCG	<1 IU/L	-
Beta-2-microglobulin	2.02 mg/L	08-2.3
IgG	8.62 g/L	7.0-16.0
IgA	1.38 g/L	0.70-4.00
IgM	1.13 g/L	0.4-2.3
Protein electrophoresis	No abnormality detected	-
Lactate dehydrogenase	549 IU/L	135-214

The laboratory findings were notable for anemia with elevated lactate dehydrogenase, suggesting a high tumor burden. Elevated D-dimer was considered nonspecific in the context of malignancy. A raised CA-125 was interpreted as a nonspecific marker of disease activity.

A multidisciplinary team, including interventional radiology, oncology, and hematology, was promptly involved. Dexamethasone was initiated immediately for suspected tumor-related edema, resulting in early improvement in the left upper-limb symptoms.

A subsequent CT-guided core biopsy of the mediastinal mass was performed and demonstrated sclerotic tissue containing atypical lymphoid infiltrates arranged in packets, with extensive areas of crush artifact. Scattered large atypical lymphoid cells with predominant nucleoli were identified without any granuloma, necrosis, or Reed-Sternberg cells. Immunohistochemical staining showed large, atypical cells that were positive for CD20, PAX5, and CD79a, confirming a B-cell lineage. The tumor cells also expressed BCL6, CD30 (patchy), MUM1, and CD23, with a high Ki-67 proliferation index. Staining was negative for CD10, Cyclin D1, TdT, ALK1, and epithelial markers (AE1/AE3), excluding germ cell tumors and thymic carcinoma. Weak polyclonal IG heavy/light chain rearrangements were found on clonality testing.

Whole-body fluorodeoxyglucose (FDG) PET-CT demonstrated a bulky anterior mediastinal mass measuring approximately 12.5 × 11.0 cm with intense heterogeneous FDG uptake (SUVmax up to 17.5). The mass encased the aortic arch, SVC, and right pulmonary artery and abutted the pericardium with a small associated pericardial effusion. A single FDG-avid pretracheal lymph node was identified. No FDG-avid disease was seen in the cervical, abdominal, or pelvic lymph nodes; bone marrow; or extranodal organs. These findings were consistent with stage IIE bulky disease. The final diagnosis was primary mediastinal large B-cell lymphoma.

The hematology team subsequently commenced R-CHOP chemotherapy (rituximab, cyclophosphamide, doxorubicin, vincristine, prednisolone), which led to progressive resolution of upper-limb congestion and complete relief of chest discomfort. She continues under hematology follow-up, and interval imaging has demonstrated a significant reduction in mediastinal mass size.

## Discussion

SVC obstruction classically presents with facial swelling, bilateral upper-limb edema, neck vein distension, and dyspnea, with symptoms typically evolving gradually as collateral venous pathways develop [1,4]. In contrast, the present case demonstrated an atypical acute presentation characterized by sudden back and central chest pain with unilateral upper-limb coldness, dusky discoloration, and delayed pulse, without the usual signs of venous congestion. Such presentations are uncommon and may closely mimic arterial emergencies, particularly aortic dissection [5].

Chest pain is a recognized but infrequent manifestation of SVC obstruction, reported in up to approximately 20% of cases [6]. Chest-predominant presentations are therefore unusual and may pose a significant diagnostic challenge. In this case, the combination of acute pain and asymmetric upper-limb vascular findings appropriately raised concern for aortic dissection, justifying urgent CT aortography. Imaging excluded arterial pathology but revealed a large anterior mediastinal mass compressing the SVC, establishing the correct diagnosis.

Histopathological examination confirmed high-grade B-cell lymphoma. Elevated lactate dehydrogenase reflected high tumor burden and aggressive disease biology [7]. Although CA-125 is traditionally associated with gynecologic malignancy, it is a nonspecific mesothelial marker and may be elevated in lymphomas, particularly in the presence of bulky disease or serosal involvement [8]. In this context, a raised CA-125 level was interpreted as a surrogate marker of disease burden rather than of gynecologic pathology.

The patient’s chest pain was unlikely to be cardiac in origin, supported by normal troponin levels and an unremarkable electrocardiogram. Instead, pain was attributed to acute venous obstruction, causing a sudden increase in mediastinal venous pressure and congestion of pain-sensitive mediastinal structures [9]. Rapid symptom resolution following dexamethasone administration further supported a venous and inflammatory mechanism rather than ischemia.

Acute SVC obstruction does not allow sufficient time for collateral venous drainage to develop, resulting in abrupt venous hypertension in affected tributaries. Compression of the left brachiocephalic vein likely caused isolated unilateral upper-limb venous congestion, accounting for the arterial-appearing clinical features. Early corticosteroid therapy reduced tumor-related edema, and timely initiation of R-CHOP chemotherapy led to significant clinical and radiological improvement.

## Conclusions

This case highlights that acute SVC obstruction can present in a markedly atypical manner. Instead of facial swelling or neck vein distension, this patient came with sudden back and chest pain and unilateral upper-limb changes that closely resembled an arterial problem. Because of this overlap, it was reasonable to initially consider aortic dissection. Early imaging proved essential, both to rule out life-threatening aortic disease and to identify the mediastinal lymphoma causing venous obstruction. This case serves as a reminder that SVC obstruction may occasionally present with atypical, localized, or pain-dominant symptoms, and clinicians should consider this possibility when assessing acute chest or back pain with limb vascular abnormalities. This case reinforces the important clinical message that acute SVC obstruction may present atypically and closely mimic arterial emergencies such as aortic dissection. Early imaging and a multidisciplinary approach are essential for prompt diagnosis and appropriate management.
